# Diagnostic Potential of Circulating CXCL13, CXCL14, and CXCL16 in Colorectal Cancer: A Preliminary Case–Control Study

**DOI:** 10.3390/cancers18193153

**Published:** 2026-09-29

**Authors:** Adrianna Romanowicz, Marta Łukaszewicz-Zając, Agnieszka Kulczyńska-Przybik, Kamil Safiejko, Marcin Juchimiuk, Barbara Mroczko

**Affiliations:** 1 Department of Neurodegeneration Diagnostics, Medical University of Bialystok, 15-089 Bialystok, Polandmroczko@umb.edu.pl (B.M.); 2 Department of Biochemical Diagnostics, University Clinical Hospital in Bialystok, 15-276 Bialystok, Poland; 3 Department of Biochemical Diagnostics, Medical University of Bialystok, 15-089 Bialystok, Poland; 4 Oncology Center of Bialystok, 15-027 Bialystok, Poland

**Keywords:** chemokines, colorectal cancer, serum biomarkers

## Abstract

**Introduction:** Epidemiological trends indicate that the global colorectal cancer (CRC) mortality will surpass 2.2 million deaths annually by the year 2050; therefore, the identification of novel circulating indicators that may support CRC diagnosis remains an important area of research. **Objectives:** The diagnostic relevance of circulating chemokines in CRC remains insufficiently understood. This study aimed to evaluate the diagnostic potential of selected serum C-X-C motif chemokines, CXCL13, CXCL14, and CXCL16, and to compare their diagnostic utility with CEA. **Patients and Methods:** This study included 62 individuals: 42 patients with CRC and 20 healthy volunteers. Serum chemokines levels were measured using a multiplex bead-based immunoassay (Luminex), and CEA levels via chemiluminescent microparticle immunoassay (CMIA). **Results:** Significant differences between patients with CRC and healthy controls were observed for serum CXCL13 concentrations. Spearman’s analysis revealed a significant negative correlation between CXCL13 and CXCL14. CXCL14 showed the highest diagnostic sensitivity among the chemokines, similar to CEA, while combining CXCL13 or CXCL14 with CEA further increased sensitivity. CXCL13 showed the highest positive predictive value (PPV) among all tested proteins, while its negative predictive value (NPV) and diagnostic accuracy (ACC) exceeded those of CXCL14 and CXCL16. CXCL13 demonstrated the highest AUC among the investigated chemokines and remained independently associated with CRC status in multivariable analysis. CXCL13 combined with CEA yielded the numerically highest overall AUC, although the improvement over CEA alone was not statistically significant. **Conclusions:** CXCL13 serves as a valuable complementary biomarker, providing crucial additive insights that might augment diagnostic frameworks of patients with CRC. These findings support further evaluation of chemokines as potential adjuncts to established diagnostic biomarkers in larger, independent screening-like study cohorts.

## Simple Summary

Colorectal cancer (CRC) remains a paramount global oncological challenge, constituting the third most prevalent malignancy worldwide. This malignancy is still frequently diagnosed at advanced stages, most often after the onset of clinical symptoms. The global CRC mortality will surpass 2.2 million deaths annually by the year 2050. Thus, given its escalating incidence, pioneering cost-effective, non-invasive diagnostic modalities focused on novel biochemical markers represent a critical clinical imperative. Although C-X-C motif chemokines have been implicated in CRC, their diagnostic significance remains insufficiently understood. This study aimed to evaluate the clinical potential of selected serum CXC chemokines, CXCL13, CXCL14, and CXCL16 in comparison to the well-established marker carcinoembryonic antigen (CEA). Based on our findings, we identify CXCL13 as the most valuable complementary biomarker among the investigated chemokines in CRC; however, independent validation is required before clinical application.

## 1. Introduction

Colorectal cancer (CRC) constitutes a major global health concern due to its high incidence and mortality. It ranks third in terms of cancer incidence worldwide and is the second most common cause of cancer-related death [1,2]. Estimates from the International Agency for Research on Cancer indicate that in 2022, approximately 1.9 million individuals were diagnosed with CRC, while nearly 904,000 deaths were attributed to this malignancy [2,3]. The development of CRC is a gradual process involving multiple stages of carcinogenesis and is often preceded by precancerous changes, particularly adenomatous polyps. According to its genetic background and pattern of inheritance, CRC can be broadly divided into sporadic, familial, and hereditary forms [4]. Understanding trends in the incidence of CRC and the clinical profile of affected patients is essential for improving diagnostic and therapeutic approaches, as well as for developing strategies aimed at predicting future changes in the CRC patient population. Greater emphasis on primary prevention, including screening programs and detection methods such as biochemical biomarkers, may contribute to lowering both the incidence of CRC and the associated mortality rate. Nevertheless, CRC is still frequently diagnosed at advanced stages, most often after the onset of clinical symptoms [5]. For this reason, the identification of novel biomarkers remains highly important. Currently, carcinoembryonic antigen (CEA) and carbohydrate antigen 19-9 (CA 19-9) are the most widely recognized serum biomarkers used in CRC diagnostics. However, their clinical utility remains limited because of insufficient diagnostic sensitivity and specificity, particularly in the early stages of disease development.

Chemokines are a heterogeneous family of small cytokines distinguished by their low molecular weight and conserved structural features. According to the arrangement of conserved N-terminal cysteine residues, they are divided into four main subfamilies: CXC, CC, CX3C, and XC [6,7]. Their biological activity is mediated through binding to specific seven-transmembrane G protein-coupled receptors (GPCRs) [8]. Importantly, individual chemokine receptors may interact with multiple ligands, while a single chemokine can activate different receptor types, creating a highly complex signaling network [9].

Chemokines are primarily involved in regulating the migration and localization of immune cells, including lymphocytes, neutrophils, eosinophils, and fibroblasts [10]. Members of the C–X–C motif (CXC) chemokine ligand family can be divided into two major groups based on the occurrence of the ELR (Glu-Leu-Arg) sequence preceding the first cysteine residue. Chemokines containing this sequence are referred to as ELR+ and include C-X-C motif chemokine ligand 1 (CXCL1) and ligand 8 (CXCL8). Their activity is mainly mediated through C-X-C chemokine receptors 1 and 2 (CXCR1 and CXCR2) and is associated with neutrophil recruitment and the regulation of angiogenesis [10,11]. Conversely, ELR− chemokines do not contain the ELR motif and exhibit different functional characteristics. This group comprises several CXC chemokines, including C-X-C motif chemokine ligand 12 (CXCL12), 13 (CXCL13), 14 (CXCL14), and 16 (CXCL16).

An increasing number of studies indicate that ELR− chemokines have a significant impact on the pathogenesis of CRC. Among them, CXCL13, CXCL14, and CXCL16 have attracted considerable attention due to their involvement in the regulation of immune cell migration, inflammatory responses, and cancer progression. CXCL13 is a small secreted chemokine that functions as a chemoattractant regulating cell migration. It exerts its biological activity by binding to C-X-C chemokine receptor type 5 (CXCR5), which triggers interactions with heterotrimeric G proteins and leads to the activation of downstream intracellular signaling cascades [12]. Elevated expression of CXCL13 has been observed in the colon under inflammatory conditions, indicating a potential contribution of this chemokine to the development and progression of CRC [13,14,15,16]. In contrast, the biological role of CXCL14 remains incompletely understood. This chemokine is expressed in numerous normal tissues and epithelial tumors, although its expression pattern is highly variable. Unlike most chemokines, its specific receptor has not yet been identified. While CXCL14 has traditionally been regarded as a tumor suppressor, recent evidence indicates that it may also support tumor progression under certain conditions [17,18,19]. CXCL16 differs from other chemokines because it exists in both transmembrane (TM-CXCL16) and soluble forms (sCXCL16) generated through cleavage by ADAM10 and ADAM17 proteases [20,21,22,23,24,25]. It is produced by macrophages, dendritic cells, monocytes, B lymphocytes, and several types of tumor cells [20,21,22]. This chemokine performs diverse biological functions. Its membrane-bound form participates in cell adhesion and acts as a scavenger receptor, whereas its soluble form functions as a chemoattractant for cells expressing the CXCR6 receptor, including activated CD8+ and CD4+ T lymphocytes as well as natural killer T (NKT) cells [23,24,25,26,27,28,29,30,31,32,33,34]. In summary, the chemokines discussed above appear to exhibit considerable potential as biomarkers of CRC.

In this study, we aimed to investigate the potential value of serum CXCL13, CXCL14, and CXCL16 as novel biomarkers for CRC and compare their diagnostic performance with that of an established biomarker—CEA. These chemokines were chosen due to the limited availability of data concerning their diagnostic relevance in CRC, despite growing evidence suggesting their involvement in cancer-related inflammation, tumor development, and metastatic dissemination. Additionally, the relationships between the serum protein concentrations and clinicopathological features of the tumor were analyzed, including TNM stage, tumor invasion depth (T), lymph node involvement (N), and the presence of distant metastases (M). The diagnostic utility of CXCL13, CXCL14, and CXCL16 was further assessed using parameters such as diagnostic sensitivity, diagnostic specificity, accuracy, positive and negative predictive values (PPVs and NPVs), and the area under the ROC curve (AUC), in comparison with the well-established CRC biomarker.

## 2. Patients and Methods

The study included 62 participants, of whom 42 were patients with CRC (26 males and 16 females, aged 33–79 years) and 20 were healthy controls (14 males and 6 females, aged 38–80 years). CRC patients were recruited at the Bialystok Oncology Centre, and the diagnosis was confirmed by histopathological evaluation of tissue samples. Disease stage was assessed using the tumor–node–metastasis (TNM) system established by the International Union Against Cancer (UICC). For further analyses, patients were categorized according to overall TNM stage and individual components of the TNM classification, including primary tumor invasion (T), regional lymph node involvement (N), and distant metastasis (M). Stage I/II disease without regional lymph node involvement was identified in 21 patients, while another 21 patients were classified as stage III/IV and had lymph node metastases. With respect to primary tumor invasion, 14 patients had T1/T2 tumors and 28 had T3/T4 tumors. Regarding distant metastases, 40 patients were classified as M0, whereas M1 disease was identified in 2 patients. Within the overall analyzed cohort, the anatomical distribution comprised 27 patients diagnosed with rectal cancer, 13 with colon cancer, and 2 with sigmoid colon cancer. In terms of histopathology, adenocarcinoma was the predominant subtype identified in 30 patients, mucinous carcinoma was presented in 3 subjects, and mixed (adenomucinous) carcinoma in the remaining 9 patients. Furthermore, the cohort exhibited high uniformity regarding tumor differentiation; the overwhelming majority (*n* = 33) presented with moderately differentiated (intermediate-grade, G2) tumors, while well-differentiated (G1, *n* = 3) and poorly differentiated (G3, *n* = 5) tumors represented a minimal fraction (with only one patient classified as GX). A total of 50 CRC patients were initially screened, 8 were excluded from the analysis due to incomplete documentation regarding tumor stage (TNM), and clinicopathological features, as these parameters are essential for accurate cohort stratification. A total of 42 patients with this malignancy were included. The clinicopathological characteristics of the study population are summarized in Table 1.

Regarding laboratory findings, only 15 (36%) patients exhibited serum C-reactive protein (CRP) levels above 10 mg/L, with no individual values exceeding 84 mg/L, thereby ruling out systemic inflammation. White blood cell (WBC) counts exceeded the reference range (4.0–10.0 × 103/µL) in only 8 (19%) patients with CRC, with all values remaining strictly below 13.13 × 103/µL.

The healthy control group comprised individuals with no prior history of chronic, systemic, or oncological diseases. To exclude active inflammatory conditions, all control participants were required to have C-reactive protein (CRP) levels below the reference threshold of 10 mg/L, which was achieved by 100% of the cohort. Exclusion criteria included pregnancy and lactation. The control cohort was strictly demographically matched to the experimental group regarding age distribution, sex ratio, and ethnicity. This study was conducted in accordance with the principles of the Declaration of Helsinki. Written informed consent was obtained from all participants prior to inclusion. The study protocol was approved by the Local Bioethics Committee (approval no. R-I-002/564/2019) of the Medical University of Bialystok. All biological samples were anonymized prior to analysis. The following exclusion criteria were applied: absence of active infection or clinical signs suggestive of infection; no history of comorbid conditions potentially influencing protein concentrations, including respiratory or gastrointestinal diseases; and no diagnosis of systemic autoimmune disorders.

Blood samples were collected from all participants before the initiation of any therapeutic intervention, including surgery, chemotherapy, radiotherapy, or other systemic treatment, to minimize the potential influence of treatment-related factors on the interpretation of serum biomarker concentrations. Samples were obtained between June 2019 and March 2021. To preserve chemokine stability, all serum samples were stored at −80 °C for a maximum duration of 24 months prior to analysis. Samples underwent no freeze–thaw cycles before the Luminex assay. Serum concentrations of CXCL13, CXCL14, and CXCL16 were determined using a multiplex bead-based assay based on Luminex technology (3-plex Luminex Human Premixed Multi-Analyte Kit, Bio-Techne R&D Systems, Minneapolis, MN, USA), following the manufacturer’s protocol. Analyte concentrations were derived from standard curves fitted using a five-parameter logistic (5-PL) model, with data processing performed using xPONENT software (version 3.1.971.0). The manufacturer-published sensitivity for the tested proteins is CXCL13—11.5 pg/mL, CXCL14—26.0 pg/mL, and CXCL16—0.40 pg/mL. The detection range for the studied parameters was as follows: CXCL13/BLC/BCA-1 12.6–3060 pg/mL, CXCL14/BRAK 97.1–23,600 pg/mL, and CXCL16 10.1–2460 pg/mL. Intra-/inter-assay CVs for CXCL13, CXCL14 and CXCL16 in our experiments were below 20%. If a measured concentration was below the lower limit of detection (LLOD), it was assigned the concentration corresponding to the lowest standard of the calibration curve for the respective analyte. Concentrations of CXCL13, CXCL14, and CXCL16 were measured simultaneously in the same multiplex Luminex assay, with the same kit lot. Samples from the study groups and reference standards were distributed across the plates in duplicates according to an identical pattern. All sample positions were randomized and assigned according to the assay plate map. A formal prospective randomization scheme was not used; therefore, we do not claim that plate-related effects were minimized by randomization. Serum carcinoembryonic antigen (CEA) concentrations were measured separately using a chemiluminescent microparticle immunoassay (CMIA) on the ARCHITECT i8200SR system (Abbott, Green Oaks, IL, USA). Optimal cut-off values determined using Youden’s index were 28.14 pg/mL for CXCL13, 1026.0 pg/mL for CXCL16, and 1.105 ng/mL for CEA. For CXCL14, an exploratory cut-off value of 554.7 pg/mL was selected to prioritize sensitivity.

### Statistical Analysis

The serum concentrations of analyzed proteins demonstrated a non-Gaussian distribution in the preliminary analysis (χ2 test); therefore, nonparametric statistical methods were applied. Differences between two independent groups were assessed with the Mann–Whitney U test, whereas associations between continuous variables were examined using Spearman’s rank correlation coefficient. The diagnostic performance of the investigated proteins was evaluated by receiver operating characteristic (ROC) curve analysis, with the corresponding areas under the curves (AUCs) calculated. Based on the biomarker-specific cut-off values, diagnostic sensitivity, specificity, and ACC were calculated. Positive predictive value (PPV) and negative predictive value (NPV) were calculated from the numbers of true-positive, false-positive, true-negative, and false-negative results obtained in the study cohort. For the combined biomarker analysis, individual biomarker results were classified as positive or negative according to their respective cut-off values. For ROC analysis of the combined biomarkers, multivariable logistic regression models were constructed, with CRC status (CRC patient vs. healthy control) as the binary dependent variable and the serum concentrations of the respective biomarkers as independent variables. The predicted probabilities obtained from the fitted logistic regression models were used to construct ROC curves and calculate AUC values for the combined biomarker models. No separate optimal cut-off values were determined for the logistic regression-based combined models, as these models were used to assess discriminatory performance based on ROC/AUC analysis. Additionally, multivariable logistic regression analysis was performed to assess the independent association of CXCL13, CXCL14, CXCL16, and CEA with CRC status. All four biomarkers were entered simultaneously into the model as continuous variables. To improve the interpretability of the effect estimate for CXCL13, CXCL13 was scaled so that the corresponding OR represented a 10 pg/mL increase in serum concentration. Multicollinearity among predictors was assessed using variance inflation factors (VIFs), and model calibration was evaluated using the Hosmer–Lemeshow goodness-of-fit test. Odds ratios (ORs) with 95% confidence intervals (CIs) and *p*-values were reported. Given the relatively small sample size, the multivariable analysis was considered exploratory. Pairwise comparisons of ROC curves were performed to assess whether differences in AUCs between CEA and the individual chemokines, as well as between CEA and the combined biomarker models, were statistically significant. Internal validation of the combined logistic regression models was performed using bootstrap resampling with 1000 repetitions. For each bootstrap sample, the logistic regression model was refitted, and its discriminatory performance was evaluated in both the bootstrap sample and the original dataset. Optimism was estimated as the mean difference between these AUC values across the bootstrap repetitions. The mean bootstrap optimism was subsequently subtracted from the apparent AUC to obtain an optimism-corrected AUC for each combined model. To account for potential optimism in data-driven cut-off selection, diagnostic performance was additionally validated for CXCL13, CXCL16, and CEA using 1000 bootstrap resamples, with the optimal cut-off re-estimated using Youden’s index in each resample and optimism-corrected estimates subsequently calculated. Microsoft Office Excel was used for the calculation of diagnostic parameters, while IBM SPSS Statistics version 20.0 and Stata/SE 19 were used for statistical analyses. Stata/SE 19 was used for bootstrap-based internal validation. GraphPad Prism version 11.0.1 for Windows (GraphPad Software, San Diego, CA, USA) was used for ROC curve analyses and graphical presentation of the results. Given the exploratory nature of the study, no formal adjustment for multiple comparisons was applied. A nominal *p*-value of <0.05 was considered statistically significant. Accordingly, findings arising from multiple comparisons should be interpreted with caution due to the increased risk of false-positive results.

## 3. Results

### 3.1. Serum Levels of CXCL13, CXCL14, CXCL16, and Well-Established Tumor Marker (CEA) in Colorectal Cancer Patients

**Figure 1:**
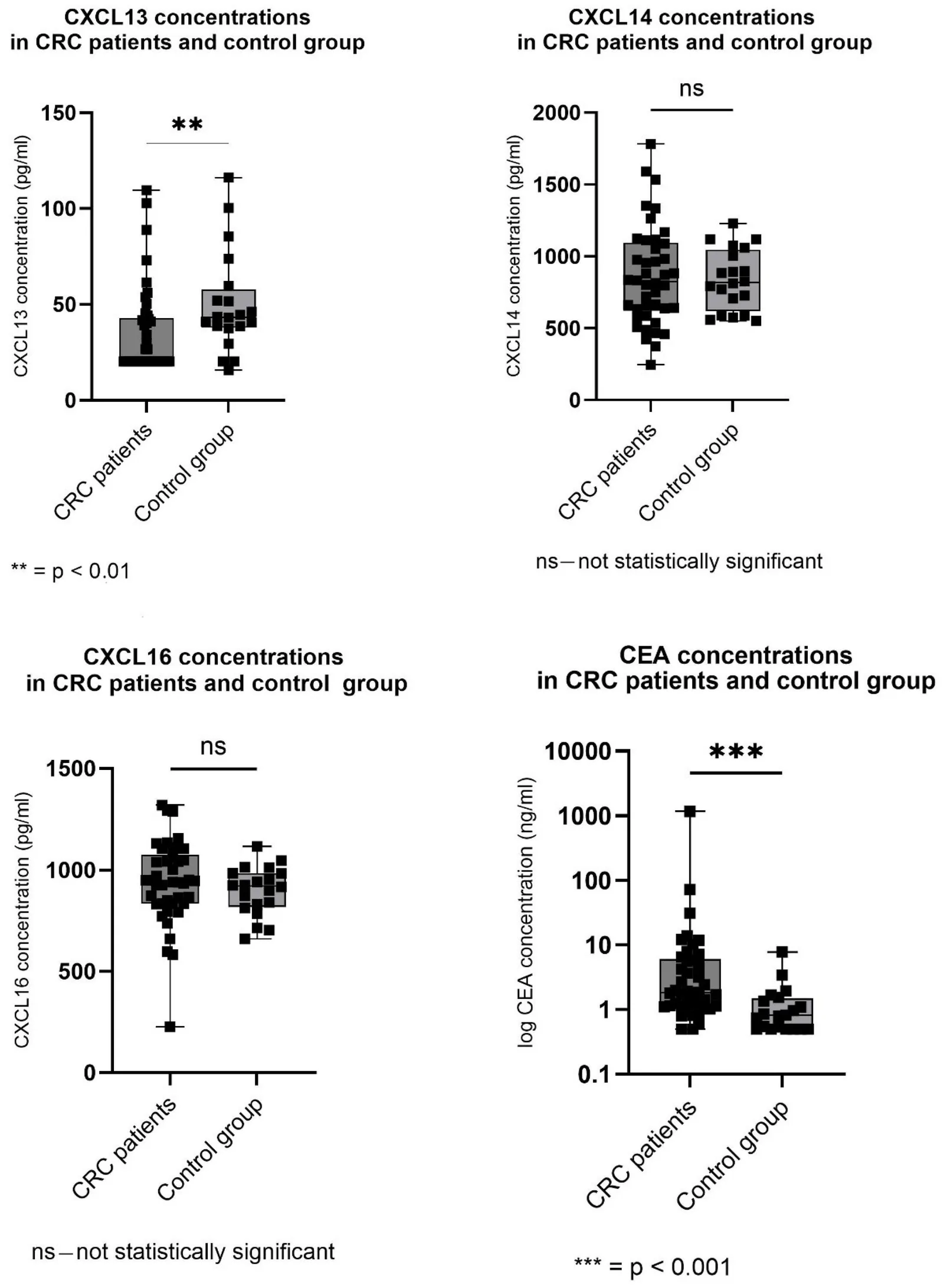
Serum concentrations of tested proteins in patients with CRC and control group.

The serum levels of CXCL13, CXCL14, CXCL16, and the classical tumor marker CEA are presented in Table 2 and Figure 1. The concentration of CXCL13 was lower, and those of CXCL14, CXCL16, and CEA were higher, in patients with CRC compared to healthy volunteers. Significant differences were observed for the levels of CXCL13 (*p* = 0.01) and CEA (*p* < 0.001) (Table 2, Figure 1).

### 3.2. Relationship Between Serum Levels of CXCL13, CXCL14, CXCL16, and CEA and Clinicopathological Features of Colorectal Cancer (CRC)

If we analyze the association between the tested proteins and tumor stage, the serum levels of CXCL13 and CXCL14 were higher in stage I/II in comparison to advanced-stage colorectal cancer (III/IV), while the concentrations of CXCL16 and CEA were found to be increased in advanced-stage CRC (Table 3). Statistically significant differences were observed only between serum CXCL13 levels at stages I/II or III/IV and healthy controls (*p* = 0.044 and *p* = 0.019, respectively). A similar association was found for CEA concentrations in stage I/II (*p* = 0.009) or III + IV (*p* < 0.001) and the healthy group (Table 4).

The assessment of the relationship between the serum concentrations of the investigated biomarkers and depth of tumor invasion (T stage) in CRC demonstrated that serum CXCL13 and CXCL 16 levels were lower while those of CXCL14 and CEA were higher in patients with T3/4-stage tumors compared to T1/T2 subjects (Table 3); however, no statistically significant differences were observed among the T1/T2 and T3/T4 subgroups. Moreover, statistically significant differences were proved for serum CXCL13 levels in T1/2 (*p* = 0.05) or T3/4 (*p* = 0.02) subgroups in relation to healthy subjects. In addition, the concentrations of CEA were significantly elevated in the T3/4 subgroup than in the control group (*p* < 0.001) (Table 4).

If we consider the presence of lymph node metastasis (N factor), the serum concentrations of CXCL13 and CXCL14 were higher in the N0 subgroup in comparison to patients with nodal involvement (N1 subgroup), whereas the serum CXCL16 and CEA levels were elevated in patients with the presence of lymph node metastasis compared to the N0 subgroup (Table 3). Statistically significant differences were revealed between serum CXCL13 as well as CEA levels in N0/N1 subgroups and healthy controls (CXCL13—*p* = 0.03 and *p* = 0.028; CEA—*p* = 0.009 and *p* < 0.001, respectively) (Table 4).

When we analyze the dependency between the presence of distant metastases (M factor) and the serum concentrations of proteins tested, CXCL16 levels were higher in patients without distant metastases compared to the M1 subgroup, opposite to the CXCL14 and CEA levels, which were elevated in the M1 subgroup in comparison to M0 patients. Given the very small number of patients in the M1 subgroup (*n* = 2), no statistical comparisons involving this subgroup were performed. Serum concentrations of CXCL13 were significantly lower in the M0 subgroup than in the control group (*p* = 0.017) (Table 4). In addition, significant associations were indicated for CEA levels in the M0 (*p* < 0.001) subgroup and healthy controls (Table 4).

Spearman’s rank correlation analysis was performed to determine the association between protein concentrations and clinicopathological parameters. No significant correlation was observed between selected CXCL levels and the baseline characteristics of patients with CRC. In contrast, a significant correlation was established between serum CEA levels and TNM stage (*p* = 0.007), and depth of tumor invasion (T factor) (*p* = 0.033). Additionally, there was a significant negative correlation between CXCL13 and CXCL14 concentrations (*p* = 0.005) (Table 5).

### 3.3. Diagnostic Usefulness of CXCL13, CXCL14, and CXCL16 in Patients with CRC Diagnosis

**Figure 2:**
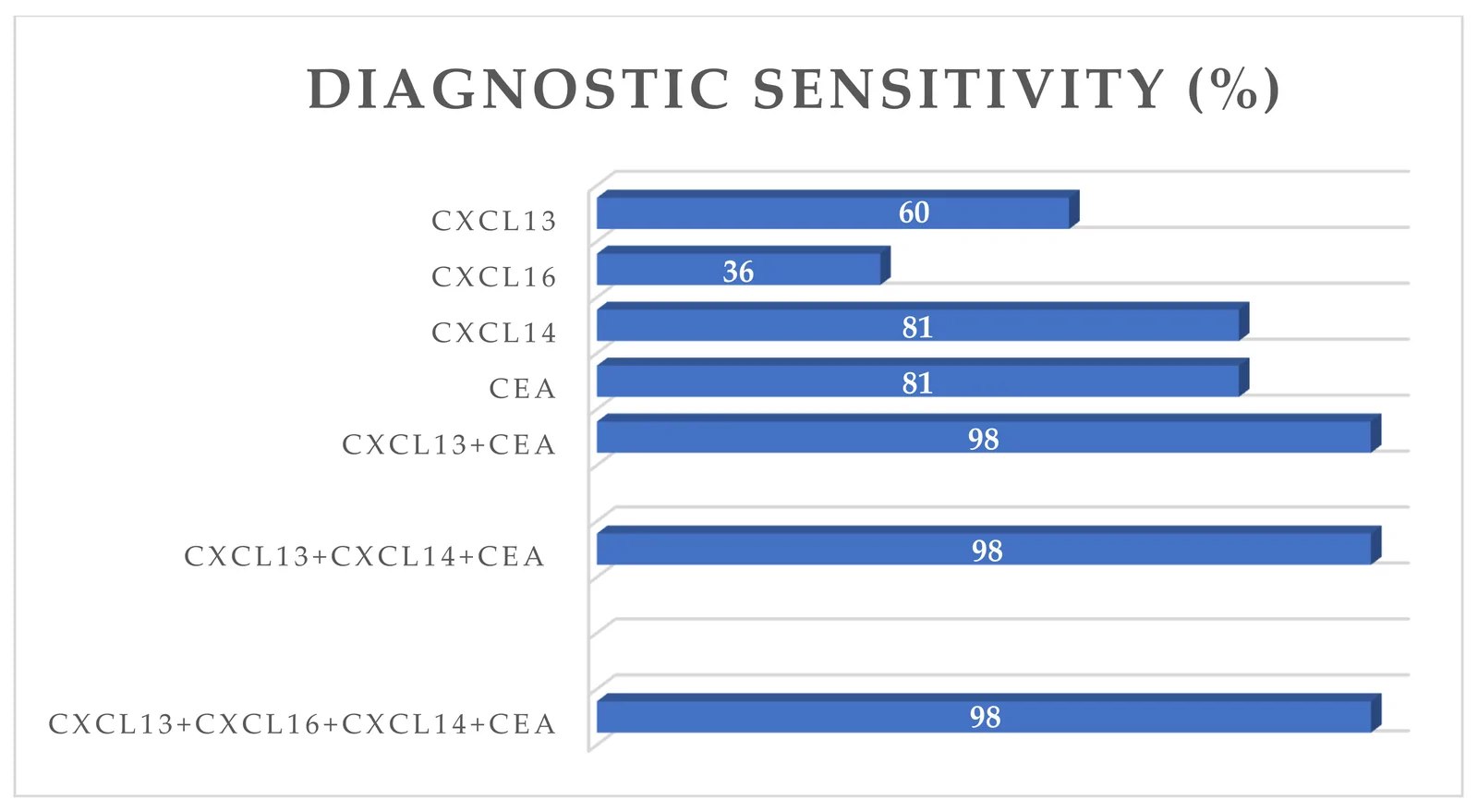
Diagnostic sensitivity of analyzed proteins.

Diagnostic criteria, including diagnostic sensitivity, diagnostic specificity, accuracy, positive and negative predictive values (PPVs and NPVs), and the areas under the ROC curve (AUC), were determined to evaluate the potential usefulness of selected chemokines in the diagnosis of CRC. We observed that among the chemokines tested (CXCL13, CXCL14, and CXCL16), the diagnostic sensitivity of CXCL14 was the highest (81%), at the exploratory cut-off of 554.7 pg/mL; however, this was accompanied by very low specificity (5%), reaching levels similar to those of the well-established tumor marker CEA (81%). The highest diagnostic sensitivity was observed for the measurement of CXCL13 or CXCL14 with CEA (both 98%) (Table 6). The combinations of all chemokines (CXC13 and CXCL14; CXCL14 and CXCL16; and CXCL13 and CXCL16) with classic tumor markers did not improve the diagnostic sensitivity (98%) (Table 7 and Figure 2). Furthermore, the diagnostic specificity of the proteins tested was also evaluated. We found that the diagnostic specificity of CXCL16 (90%) was higher than that of other analyzed chemokines as well as notably elevated compared to that of the well-established tumor marker for CRC—CEA (70%) (Table 6). The positive predictive value (PPV) for the measurement of CXCL13 levels (89%) was higher among all proteins tested, including the classic tumor marker CEA (85%). The negative predictive value (NPV) of CXCL13 was also higher than that of CXCL14 and CXCL16, but lower in comparison to that of CEA. A similar trend was observed regarding diagnostic accuracy (ACC); CXCL13 demonstrated the highest ACC among all analyzed chemokines (68%), although this diagnostic criterion remained lower than that of CEA (77%) (Table 6). Bootstrap-based optimism correction resulted in modest reductions in the diagnostic performance estimates for CXCL13, CXCL16, and CEA. For CXCL13, the apparent sensitivity, specificity, PPV, NPV, and accuracy were 59.5%, 85.0%, 89.3%, 50.0%, and 67.7%, respectively, compared with optimism-corrected values of 58.1%, 81.4%, 87.3%, 47.5%, and 65.5%. For CXCL16, the corresponding apparent estimates were 35.7%, 90.0%, 88.2%, 40.0%, and 53.2%, and the optimism-corrected values were 32.8%, 83.8%, 83.6%, 35.7%, and 49.1%, respectively. For CEA, the apparent estimates of 81.0%, 70.0%, 85.0%, 63.6%, and 77.4% were reduced to 78.6%, 65.0%, 82.7%, 59.4%, and 74.3% after optimism correction, respectively.

**Figure 3:**
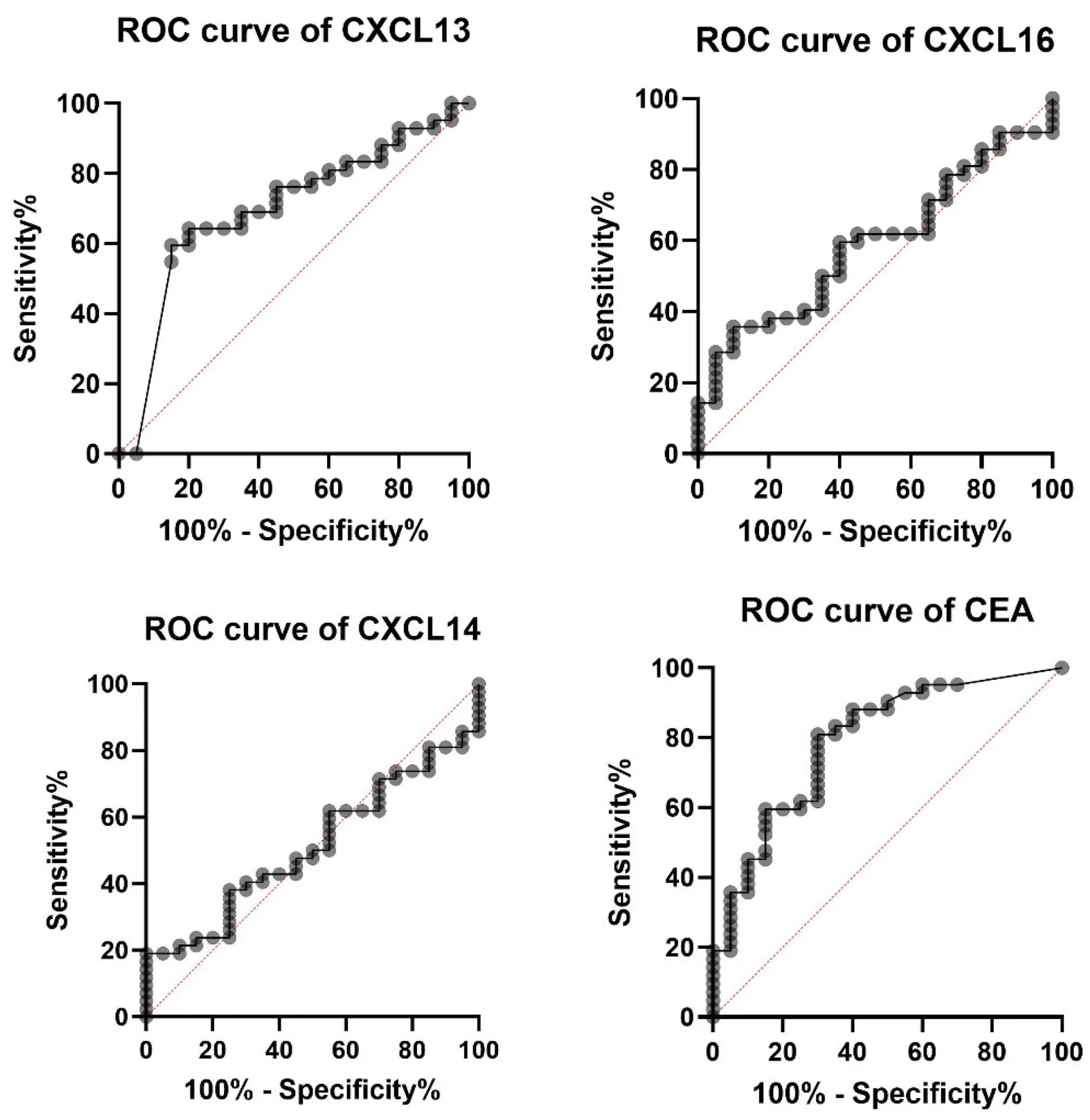
Area under the ROC curves of analyzed proteins (AUC CXCL13 = 0.6964, (95% CI: 0.5546–0.8382); AUC CXCL14 = 0.5107, (95% CI: 0.3658–0.6556); AUC CXCL16 = 0.5880, (95% CI: 0.4449–0.7313); and AUC CEA = 0.7911, (95% CI: 0.669–0.9131). Formal ROC curve comparisons: CEA vs. CXCL13, *p* = 0.379; CEA vs. CXCL14, *p* = 0.006; and CEA vs. CXCL16, *p* = 0.018.

**Figure 4:**
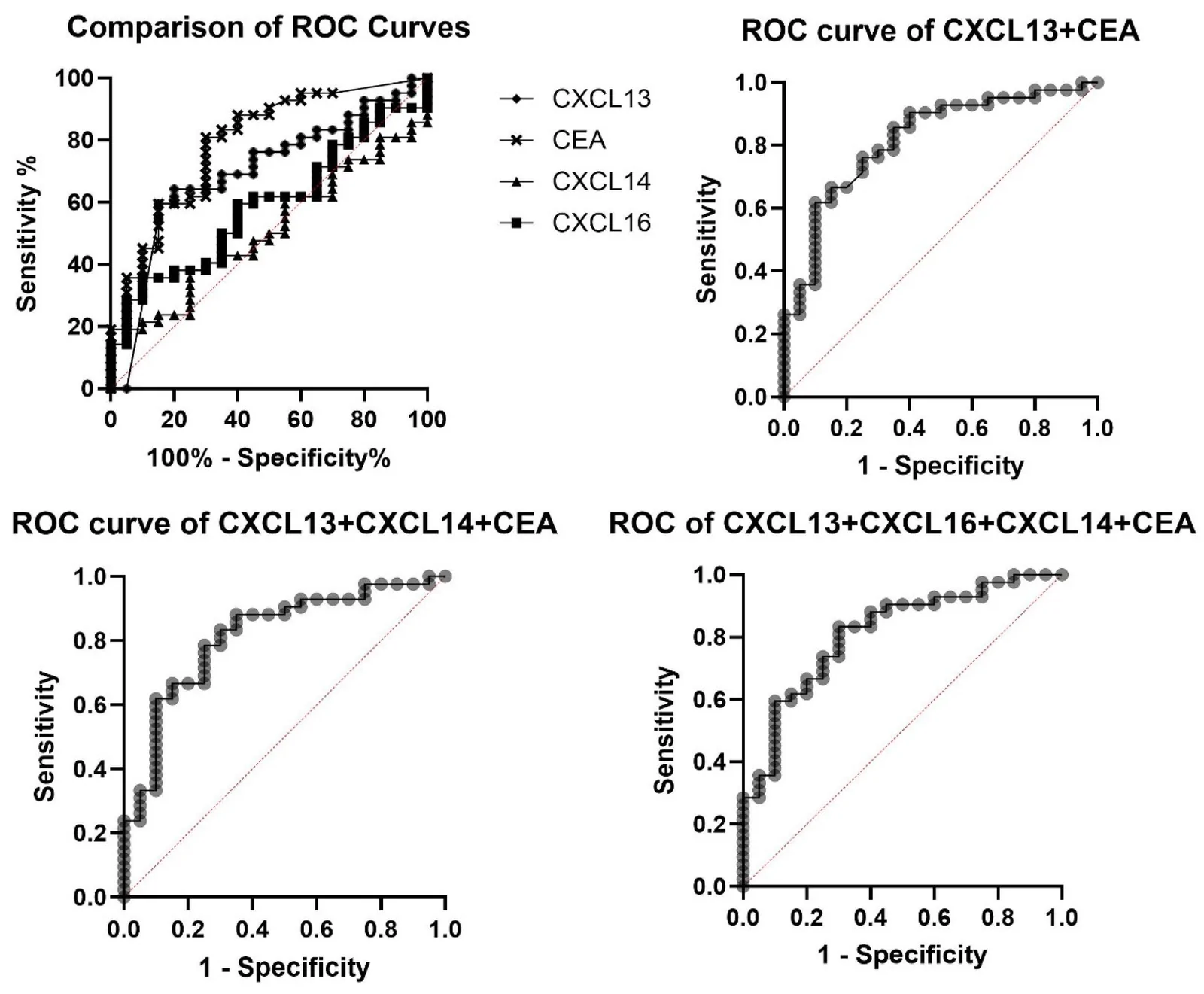
Area under the ROC curves of combined proteins (AUC CXCL13 + CEA = 0.819; (95% CI: 0.7071–0.9310); AUC CXCL13 + CXCL14 + CEA = 0.816, (95% CI: 0.7014–0.9295); AUC CXCL13 + CXCL16 + CXCL14 + CEA = 0.813, 95% CI: 0.7007–0.9255). Formal ROC curve comparisons versus CEA: CXCL13 + CEA, *p* = 0.645; CXCL13 + CXCL14 + CEA, *p* = 0.694; and CXCL13 + CXCL14 + CXCL16 + CEA, *p* = 0.719.

The area under the ROC curve (AUC) was used to assess the diagnostic performance of the studied proteins. Among the individual chemokines, CXCL13 demonstrated the highest AUC of 0.6964 (95% CI: 0.5546–0.8382), compared with CXCL14 (AUC = 0.5107; 95% CI: 0.3658–0.6556) and CXCL16 (AUC = 0.5880; 95% CI: 0.4449–0.7313). CEA demonstrated an AUC of 0.7911 (95% CI: 0.6690–0.9131) (Figure 3). Formal ROC curve comparisons revealed significantly higher AUC for CEA compared with CXCL14 (*p* = 0.006) and CXCL16 (*p* = 0.018), while no significant difference was observed between CEA and CXCL13 (*p* = 0.379). The combination of CXCL13 with CEA yielded the highest AUC (0.8190; 95% CI: 0.7071–0.9310) (Figure 4). However, the observed numerical increases in AUC for the combined models compared with CEA alone did not reach statistical significance (CXCL13 + CEA: *p* = 0.645; CXCL13 + CXCL14 + CEA: *p* = 0.694; and CXCL13 + CXCL14 + CXCL16 + CEA: *p* = 0.719). Internal validation using 1000 bootstrap resamples demonstrated an optimism-corrected AUC of 0.804 for the CXCL13 + CEA model, compared with an apparent AUC of 0.819. The estimated optimism was 0.015. For the CXCL13 + CXCL14 + CEA model, the apparent AUC was 0.816, with an estimated optimism of 0.032, resulting in an optimism-corrected AUC of 0.784. For the CXCL13 + CXCL14 + CXCL16 + CEA model, the apparent AUC was 0.813, and the estimated optimism was 0.047, yielding an optimism-corrected AUC of 0.766. Moreover, multivariable logistic regression analysis was performed to assess the independent association of the analyzed biomarkers with CRC status. The overall model including CXCL13, CXCL14, CXCL16, and CEA was statistically significant (LR χ2(4) = 15.77, *p* = 0.0033). CXCL13 remained significantly associated with CRC status (OR per 10 pg/mL increase = 0.741; 95% CI: 0.564–0.974; *p* = 0.031), whereas CXCL14 (OR = 1.000; 95% CI: 0.997–1.002; *p* = 0.715), CXCL16 (OR = 1.001; 95% CI: 0.997–1.005; *p* = 0.643), and CEA (OR = 1.518; 95% CI: 0.987–2.335; *p* = 0.058) were not statistically significant in the multivariable model. No evidence of problematic multicollinearity among the predictors was observed, with VIF values ranging from 1.03 to 1.12 (mean VIF = 1.08). The Hosmer–Lemeshow goodness-of-fit test showed no evidence of significant lack of fit (χ2(8) = 5.88, *p* = 0.661).

## 4. Discussion

Colorectal cancer (CRC) remains a paramount global oncological challenge, constituting the third most prevalent malignancy worldwide. Given its escalating incidence, pioneering cost-effective, non-invasive diagnostic modalities focused on novel biochemical markers represent a critical clinical imperative. Current pathophysiological insights indicate that specific chemokines act as key regulators in tumorigenesis and the metastatic cascade. Notably, C-X-C chemokine motifs have been shown to modulate tumor-induced angiogenesis and drive distant metastasis in multiple malignancies, including colorectal adenocarcinoma. Among them, CXCL13, CXCL14, and CXCL16 have attracted considerable attention due to their involvement in the regulation of immune cell migration, inflammatory responses, and cancer progression. Recently, in our review paper we presented the potential of selected C-X-C motif chemokines as biomarkers in CRC diagnosis [35,36,37,38]. Based on this systemic review, we concluded that chemokines and receptors are promising blood biomarkers and therapeutic targets in CRC. Therefore, this study extends our prior investigations into the clinical utility of chemokines as potential oncological biomarkers in gastrointestinal tracts, including gastric, pancreatic, and colorectal cancer [35,36,37]. While the multifunctional roles of CXCL13, CXCL14, and CXCL16 in CRC progression are increasingly characterized, little is known about their diagnostic efficacy as candidate CRC biomarkers in direct comparison with the well-established, first-line tumor marker CEA.

In this study, we found that the serum levels of CXCL13 were significantly lower in patients with CRC than in healthy volunteers. A significant difference was also observed for the classical tumor marker CEA. In accordance to our findings, other evidence also suggests that CXCL13 together with its specific receptor—CXCR5—might play potential roles in regulating tumor occurrence, progression, metastasis, and prognosis in the microenvironment where the tumor is located [39,40,41,42,43]. Zhang et al. [44] assessed serum CXCL13 levels in 5-Fu (5-fluorouracil)-sensitive and 5-Fu-resistant patients using an enzyme-linked immunosorbent assay (ELISA). The investigators proved that CXCL13 was highly expressed in 5-Fu-resistant patients compared to 5-Fu-sensitive patients [44]. Moreover, contrary to our findings, the concentration of secreted CXCL13 proteins was significantly elevated in both resistant CRC cell lines compared to the supernatant of cell cultures of non-resistant cell lines [44]. CXCL13 released through the M2 polarization of macrophages activates the CXCL13/CXCR5 axis in tumor cells and stimulates CRC liver metastasis, promoting 5-FU chemoresistance [44]. Zhu et al. [45] demonstrated that CXCL13 potentiates the proliferation and migratory capacity of CRC cells through the oncogenic activation of the PI3K/Akt signaling cascade. Consistent with these findings, Qi et al. [46] reported significant upregulation of both CXCL13 and its cognate receptor, CXCR5, in malignant colorectal tissues relative to adjacent non-neoplastic mucosa. Moreover, in our study, the serum levels of CXCL13 were higher in I/II CRC in comparison to advanced colorectal cancer, while statistically significant differences were obtained only between serum CXCL13 and CEA levels in both of the analyzed subgroups compared to healthy controls. In addition, serum CXCL13 levels were lower in patients with stage T3/4 tumors compared to T1/T2 subjects, and statistically significant differences were observed for serum CXCL13 levels in both subgroups, while CEA levels were only significant in the T3 + T4 subgroup compared to the control group. Serum concentrations of CXCL13 were higher in the N0 subgroup in comparison to patients with nodal involvement, while statistically significant differences were observed between serum CXCL13 and CEA levels in both subgroups and healthy controls. Data regarding circulating CXCL13 levels in patients with CRC, particularly in relation to disease advancement, remain limited. Therefore, most available evidence concerning the association between CXCL13 and CRC progression is derived from studies evaluating its expression in tumor tissue [44]. Opposite to our results, the expression of CXCL13 was noticeably increased in CRC tissues compared with adjacent cancer cells, which was more apparent in tumors with higher tumor staging [44]. Luo et al. also found that CXCL13 was sufficiently expressed in CRC tissues and positively correlated with clinicopathological features such as more advanced TNM stage, a greater depth of tumor invasion and the presence of lymph node metastases [47]. Some recent studies have found that CXCL13 has a predictive value for the prognosis of patients with CRC [44,48]. The findings of Tan et al. [49] have evaluated the relationship between necroptosis-related genes (NRGs) including *CXCL13* and CRC development. The authors reported that NRGs were proved to be an accurate predictor of CRC prognosis, which may confirm the role of CXCL13 in CRC pathogenesis [49]. If we take into consideration the serum levels of CXCL14 and CXCL16, the results of our study failed to indicate statistically significant differences between serum levels of CXCL14, CXCL16, and CEA in patients with CRC and healthy volunteers. In addition, serum CXCL14 levels were elevated in I/II CRC compared with advanced CRC, whereas the concentrations of CXCL16 and CEA were higher in advanced-stage CRC; however, these differences were not significant. The serum concentrations of CXCL14 were elevated in the less advanced CRC (T1/T2 and N0 subgroups), opposite to CXCL16 and CEA. These observations are supported by previous studies of other investigators reporting upregulated CXCL14 levels in CRC [50]. Zhang et al. [50] revealed a negative correlation between CXCL14 expression and the stage of colon cancer, consistent with previous studies on the epigenetic silencing of CXCL14 [51] using the cBioPortal database. Moreover, common human and mouse CRC cell lines have also exhibited almost complete silencing of cancer cells, indicating that CXCL14 is inversely associated with tumor proliferation [50]. Overexpressing CXCL14 directly suppresses colon cancer cell proliferation, migration, and metastasis by blocking matrix metalloproteinases (MMPs), which facilitate tumor invasion into surrounding tissues and lymphatic vessels [50]. The authors have concluded that CXCL14 inhibits colon cancer development through two primary mechanisms—the suppression of tumor cell proliferation, migration, and metastasis and the remodeling of the immune microenvironment—indicating that CXCL14 may represent a potential target for immunotherapy [50]. Conversely, the downregulation of this parameter has also been documented. Lin et al. [52], for instance, utilized quantitative PCR and immunohistochemistry to confirm a marked downregulation of CXCL14 in CRC tissue compared to normal mucosa. Similarly, Cao et al. [53] reported promoter hypermethylation and subsequent silencing of CXCL14 across five CRC cell lines. Furthermore, Zeng et al. [54] demonstrated that CXCL14 drives the proliferation and migration of reactive oxygen species (ROS)-induced CRC cells, noting elevated chemokine levels following H2O2 exposure. These phenotypic discrepancies warrant careful consideration and align with discordances noted by other investigators [55]. In contrast to our study, other researchers using the IHC method reported that CXCL14 expression significantly correlated with TNM stage, histodifferentiation, and tumor size [56]. It is widely hypothesized that the biological impact of CXCL14 might be determined by the local cellular environment, tissue-specific architecture, and interacting protein cascades. For example, while tumor growth is typically suppressed by CXCL14 originating from epithelial structures, the chemokine acts as an oncogenic and metastatic driver when secreted by cancer-associated fibroblasts (CAFs). Restoring CXCL14 expression effectively suppressed CRC cell proliferation, migration, invasion, and epithelial-to-mesenchymal transition (EMT) [53]. These insights underscore CXCL14 as a potential therapeutic target, suggesting that elevated systemic levels of this chemokine may serve as a favorable prognostic biomarker in CRC. Moreover, given these dual roles, further mechanistic and clinical validation is highly warranted [44]. Regarding CXCL16, our analysis revealed no statistically significant differences in serum concentrations between the cohorts; however, a trend toward elevated levels was observed in the study group compared to healthy controls. In contrast, numerous previous studies [29,57,58,59,60,61,62] have consistently reported significantly increased circulating CXCL16 concentrations, as well as elevated tissue and mRNA expression, in patients with CRC and CRC cell lines compared with controls. Furthermore, Chen et al. [62] reported that CXCL16-positive tissue expression correlates significantly with tumor size, histological differentiation, and distant metastasis. Despite the lack of statistical significance, our findings indicate a trend consistent with previous reports. A larger cohort may improve statistical power and enable the confirmation of these observations throughout all CRC stages. The functional profile of CXCL16 in advanced malignancy appears highly diverse. Some studies have demonstrated that CXCL16 may promote tumor-associated angiogenesis following minimally invasive colorectal surgery [63], whereas others suggest it may suppress the development of liver metastases [64]. The anti-metastatic potential of CXCL16 was further supported by experimental studies demonstrating that CXCL16 expression enhances tumor necrosis factor-alpha (TNF-α)-induced apoptosis through the activation of the caspase-3/PARP pathway and inhibition of NF-κB-mediated (nuclear factor kappa B) survival signaling. Moreover, CXCL16 promoted the accumulation of M1 macrophages within metastatic liver lesions, resulting in increased TNF-α production and suppression of colorectal cancer liver metastasis. These findings suggest that CXCL16 may exert protective effects in CRC progression by simultaneously enhancing anti-tumor immune surveillance and inducing apoptosis of malignant cells, highlighting the complex and context-dependent biological role of this chemokine in cancer development [65], including colorectal carcinogenesis [66]. In our previous studies, we indicated elevated serum levels of other CXC chemokines, such as CXCL8 in patients with CRC [37]. We found that serum concentrations of CXCL8, similarly to those of CEA, were significantly higher in patients with CRC than in healthy controls. Moreover, there were statistically significant differences in CXCL8 concentrations between tumor stages. CXCL8 levels were also significantly elevated in patients with CRC with distant metastases compared to patients in the subgroup without metastases. We concluded that serum CXCL8 might be a potential biomarker of CRC progression [37]. In this study, Spearman’s rank correlation analyses revealed no significant correlation between selected CXCL levels and the baseline characteristics of patients with CRC; however, CXCL13 demonstrated a significant correlation with CXCL14 levels. In addition, a significant association was established between serum CEA levels and TNM stage and depth of tumor invasion. Some clinical investigations have indicated that CXCL16 is significantly correlated with TNM stage and CEA levels, whereas no correlations were proved for CXCL14 concentrations [67]. Given the absence of comparable research concerning Spearman’s rank correlation analyses of CXCL13 levels, as a preliminary study, our work highlights a less explored area of research and demonstrates the need for further investigation in a larger patient cohort.

The investigated chemokines showed different diagnostic performance profiles. CXCL16 demonstrated the highest specificity among the analyzed chemokines, whereas CXCL13 showed the highest PPV and accuracy. Although CXCL14 showed relatively high sensitivity at the exploratory sensitivity-oriented cut-off, this was accompanied by very low specificity, substantially limiting its standalone diagnostic performance. The NPV of CXCL13 was higher than those of CXCL14 and CXCL16 but lower than that of CEA. Combining CXCL13 or CXCL14 with CEA numerically increased diagnostic sensitivity. In addition, among the analyzed chemokines, CXCL13 demonstrated the highest AUC, although its discriminatory performance did not differ significantly from that of CEA. The combination of CXCL13 with CEA yielded a numerically higher AUC than CEA alone; however, this increase was not statistically significant. Bootstrap-based internal validation showed only a small reduction in the AUC of the CXCL13 + CEA model, supporting the stability of its estimated discriminatory performance. Furthermore, in multivariable logistic regression analysis, CXCL13 remained independently associated with CRC status, whereas CXCL14 and CXCL16 were not statistically significant and CEA showed only a borderline association. These findings suggest that, among the investigated chemokines, CXCL13 may provide diagnostic information independently of the other biomarkers included in the multivariable model. Similar results were found in our previous study, where we compared the diagnostic criteria of serum CXCL8 levels with the classical tumor marker for CRC (CEA). The diagnostic sensitivity, NPV, and ACC were higher for serum CXCL8 than for CEA [37]. Furthermore, the AUC for CXCL8 was higher than for CEA in patients with CRC [37].

This study has several limitations that warrant consideration. The relatively small sample size may constrain statistical power and the precision of diagnostic performance estimates, thereby limiting the generalizability of the findings. This constraint was partly related to a stringent selection strategy designed to minimize potential confounding factors. To reduce the potential influence of systemic factors on circulating chemokine profiles, we exclusively enrolled treatment-naïve patients and excluded individuals with active inflammatory, autoimmune, or comorbid conditions. While this strict approach limited the recruitment rate, it allowed for the evaluation of circulating chemokine concentrations in a more homogeneous study population. Therefore, the present study should be considered exploratory and hypothesis-generating, and its findings require confirmation in larger, independent cohorts. Additionally, unlike CXCL13, CXCL16, and CEA, the cut-off for CXCL14 was selected using an exploratory sensitivity-oriented approach rather than by maximizing Youden’s index, which should be considered when interpreting its diagnostic performance. Moreover, the control group consisted of healthy individuals rather than patients with clinically relevant non-malignant gastrointestinal conditions. Consequently, the present findings demonstrate discrimination between patients with CRC and healthy controls but do not establish the ability of the investigated chemokines to distinguish CRC from non-malignant gastrointestinal diseases. Furthermore, the PPV and NPV reported in this study should be interpreted with caution, as these measures are prevalence-dependent and reflect the specific proportion of patients with CRC and healthy controls in the present cohort. Therefore, they should be regarded as cohort-dependent estimates and should not be directly extrapolated to screening settings, the general population, or other clinical populations with a different CRC prevalence. Moreover, although all serum samples underwent only a single freeze–thaw cycle prior to analysis to mitigate pre-analytical confounding factors, the inherently labile nature of these biomarkers remains a concern. This investigation focused exclusively on quantifying circulating CXCL concentrations, without parallel evaluation of tumor tissue expression at either the mRNA or protein level. Therefore, circulating concentrations cannot be assumed to directly reflect tumor tissue expression or to establish the tissue source or biological mechanisms underlying the observed differences. To evaluate the potential diagnostic utility of selected CXCLs in CRC, a multiplex bead-based immunoassay utilizing Luminex technology was employed. While the Luminex platform has inherent technical limitations, including potential matrix interference, background noise, antibody cross-reactivity, and batch-to-batch variability, its multiplex capabilities enabled the concurrent quantification of multiple CXCL family chemokines within a single reaction well. In addition, the solution-phase kinetics of suspended microparticles facilitate antigen–antibody interactions, while the broad dynamic range enables the concurrent measurement of analytes across different concentration ranges. Thus, the Luminex platform provided an appropriate multiplex approach for the biomarker profiling performed in the present exploratory study. Given the exploratory, single-center design of the present study and the lack of an available independent cohort, validation was focused, at this stage, on an internal bootstrap approach. The combined diagnostic models were therefore validated using 1000 bootstrap repetitions with optimism correction to assess the robustness of their diagnostic performance and reduce the risk of overestimation. However, internal validation cannot substitute for evaluation in an independent cohort. External validation in larger, independent and clinically representative cohorts, including patients with non-malignant gastrointestinal conditions, represents an important next step. The present findings should therefore be regarded as hypothesis-generating and as a basis for future multicenter studies aimed at determining the potential clinical utility of these biomarkers.

Clinical heterogeneity severely limits the utility of chemokines as novel CRC biomarkers. Given their highly pleiotropic nature, chemokine expression profiles shift dynamically from tumor suppression in the early stages of the disease to metastasis promotion in advanced stages. This functional duality is further compounded by molecular divergence among consensus molecular subtypes (CMSs); for instance, a biomarker for the immunoactive CMS1 (MSI) subtype fails to detect mesenchymal CMS4 tumors. Consequently, analyzing the levels of specific chemokines in CRC patients is crucial—an approach we adopted in our preliminary study, which will serve as the foundation for future, larger-scale research. Furthermore, since chemokines mediate systemic inflammation, common comorbidities such as IBD or metabolic syndrome can trigger systemic cross-reactivity, leading to a high rate of false-positive results. Finally, pronounced intratumoral heterogeneity—specifically between the tumor core and the invasive front—can lead to biopsy errors, while inter-patient baseline differences driven by gut microbiota make it impossible to establish universal cutoff values. To address this, we matched our CRC patient cohort with a control group of healthy individuals based on age, sex, race, and demographic characteristics, while also excluding subjects with active inflammation or metabolic conditions such as diabetes.

Taken together, these findings indicate that CXCL13 shows the most promising diagnostic potential among the investigated chemokines. Importantly, the independent association of CXCL13 with CRC status in the multivariable model further supports its potential diagnostic relevance. Therefore, CXCL13 may represent a potential complementary biomarker to the well-established tumor marker CEA; however, further validation in larger, independent cohorts is required to confirm its clinical utility. Except for our previous work, no other published studies have similarly evaluated these parameters in this manner.

## 5. Conclusions

The increasing burden of CRC highlights the need for improved diagnostic approaches that may support its detection. Although CXC motif chemokines have been implicated in CRC, the significance of serum CXCL13, CXCL14, and CXCL16 concentrations remains insufficiently understood. In the present study, among the analyzed chemokines, CXCL13 demonstrated the highest diagnostic potential, while multivariable logistic regression analysis further showed that CXCL13 remained independently associated with CRC status. In conclusion, while not positioned as a standalone superior biomarker, CXCL13 provides critical complementary information that significantly enhances overall diagnostic significance. Given the exploratory and hypothesis-generating nature of the present study, these findings require independent validation. Further studies in larger and clinically representative cohorts, including patients with non-malignant gastrointestinal conditions, are needed before the potential clinical application of CXCL13 and the other investigated chemokines as biomarkers can be considered.

## Figures and Tables

**Table 1:** Characteristics of patients.

Variable Tested		Number of Patients
Group	Colorectal cancer	42
	Control (healthy controls)	20
Age (range and median)	Colorectal cancer	33–79, median 63
	Control (healthy controls)	38–80, median 55
Gender	Colorectal cancer	26 males and 16 females
	Control (healthy controls)	14 males and 6 females
TNM (tumor–nodulus–metastasis) stage	I + II	21
	III + IV	21
Nodal involvement (N factor)	N0	21
	N1	21
Distant metastases (M factor)	M0	40
	M1	2
Depth of tumor invasion (T factor)	T1 + T2	14
	T3 + T4	28
Degree of differentiation (G factor)	Well-differentiated, G1	3
	Intermediate-grade, G2	33
	Poorly differentiated, G3	5
	Tumor grade could not be assessed, Gx	1
Tumor location	Rectal cancer	27
	Colon cancer	13
	Sigmoid colon cancer	2
Histological subtype	Adenocarcinoma	30
	Mucinous carcinoma	3
	Adenomucinous	9

**Table 2:** Serum levels of proteins tested in patients with CRC in comparison to healthy controls.

		CXCL13 (pg/mL)	CXCL16 (pg/mL)	CXCL14 (pg/mL)	CEA (ng/mL)
Colorectal cancer patients	Mean	35.12	937.70	860.58	33.62
	Std. Deviation	23.04	203.95	344.13	181.04
	Median	20.3	943.86	824.175	1.84
Healthy group	Mean	49.98	897.00	839.06	1.36
	Std. Deviation	26.06	120.15	210.90	1.68
	Median	43.35	920.58	818.9	0.81
*p* (Mann–Whitney test)		0.01 *	0.27	0.90	<0.001

* Statistically significant when *p* < 0.05.

**Table 3:** Serum levels of proteins tested in colorectal cancer patients in relation to tumor stage and clinicopathological features of tumor.

		CXCL13 (pg/mL)	CXCL16 (pg/mL)	CXCL14 (pg/mL)	CEA (ng/mL)
TNM stages I and II	Mean	34.925	908.043	826.301	3.887
	Std. Deviation	17.952	167.291	360.465	6.852
	Median	29.76	933.75	834.480	1.270
TNM stages III and IV	Mean	35.313	967.360	894.855	63.352
	Std. Deviation	27.681	235.450	332.219	255.513
	Median	20.30	970.60	813.870	3.170
*p* (Mann–Whitney test)		0.329	0.097	0.546	0.059
T1 + T2	Mean	35.087	920.105	811.732	4.453
	Std. Deviation	24.630	214.637	440.997	8.186
	Median	23.480	945.940	648.770	1.270
T3 + T4	Mean	35.135	946.500	885.001	48.203
	Std. Deviation	22.678	201.844	290.409	221.532
	Median	20.300	943.285	854.200	2.215
*p* (Mann–Whitney test)		0.850	0.679	0.169	0.060
N0	Mean	34.212	907.361	842.642	3.887
	Std. Deviation	17.357	167.181	350.640	6.852
	Median	29.760	926.100	834.480	1.270
N1	Mean	36.025	968.043	878.514	63.352
	Std. Deviation	28.029	235.345	345.181	255.513
	Median	20.300	970.560	813.870	3.170
*p* (Mann–Whitney test)		0.433	0.083	0.841	0.060
M0	Mean	35.860	939.247	861.591	34.860
	Std. Deviation	23.380	208.769	352.679	185.532
	Median	20.300	943.860	824.175	1.825
M1	Mean	20.300	906.800	840.325	8.810
	Std. Deviation	0.000	59.991	60.479	7.283
	Median	20.300	906.800	840.325	8.810

**Table 4:** Statistical differences between different stratified groups (TNM stage; factors T, N, and M) and healthy controls.

		CXCL13 (pg/mL)	CXCL16 (pg/mL)	CXCL14 (pg/mL)	CEA (ng/mL)
TNM stage I + II	*p* (Mann–Whitney test)	0.044 *	0.927	0.686	0.009 *
TNM stage III + IV	*p* (Mann–Whitney test)	0.019 *	0.070	0.865	<0.001 *
T1 + T2	*p* (Mann–Whitney test)	0.050 *	0.637	0.255	0.058
T3 + T4	*p* (Mann–Whitney test)	0.020 *	0.213	0.623	<0.001 *
N0	*p* (Mann–Whitney test)	0.030 *	0.927	0.865	0.009 *
N1	*p* (Mann–Whitney test)	0.028 *	0.070	0.969	<0.001 *
M0	*p* (Mann–Whitney test)	0.017 *	0.249	0.894	<0.001 *

* Statistically significant when *p* < 0.05 in Mann–Whitney test.

**Table 5:** Correlations between serum CXCL13, CXCL16, CXCL14, and CEA levels and clinicopathological parameters in colorectal cancer patients.

Variable	CXCL13	CXCL16	CXCL14	CEA	TNM	T	N
**CXCL13**	1.000	0.037 (0.818)	**−0.426 (0.005)**	0.066 (0.676)	−0.108 (0.497)	−0.072 (0.651)	−0.125 (0.432)
**CXCL16**	—	1.000	0.019 (0.903)	0.277 (0.076)	0.247 (0.114)	0.060 (0.705)	0.273 (0.080)
**CXCL14**	—	—	1.000	0.111 (0.484)	0.102 (0.520)	0.232 (0.139)	0.033 (0.834)
**CEA**	—	—	—	1.000	**0.410 (0.007)**	**0.330 (0.033)**	0.297 (0.056)
**TNM**	—	—	—	—	1.000	**0.523 (<0.001)**	**0.813 (<0.001)**
**T**	—	—	—	—	—	1.000	0.172 (0.275)
**N**	—	—	—	—	—	—	1.000

Data are presented as Spearman’s rank correlation coefficient (ρ), with the corresponding *p*-value in parentheses. Statistically significant correlations (*p* < 0.05) are shown in bold.

**Table 6:** Apparent diagnostic performance of the analyzed proteins in the study cohort.

	Diagnostic Sensitivity (%)	Diagnostic Specificity (%)	Positive Predictive Value (PPV) (%)	Negative Predictive Value (NPV) (%)	Diagnostic Accuracy (ACC) (%)
CXCL13	60	85	89	50	68
CXCL16	36	90	88	40	53
CXCL14	81	5	64	11	56
CEA	81	70	85	64	77

**Table 7:** Diagnostic sensitivity of the combined proteins.

	Diagnostic Sensitivity (%)
CXCL13 + CEA	98
CXCL14 + CEA	98
CXCL16 + CEA	88
CXCL13 + CXCL16	71
CXCL13 + CXCL14	80
CXCL14 + CXCL16	91
CXCL13 + CXCL16 + CEA	98
CXCL13 + CXCL14 + CEA	98
CXCL14 + CXCL16 + CEA	98
CXCL13 + CXCL14 + CXCL16	91
CXCL13 + CXCL14 + CXCL16 + CEA	98

## Data Availability

Data will be made available on request, due to privacy/ethical restrictions.
